# Supplementary material for: A Global Screen for Assembly State Changes of the Mitotic Proteome by SEC-SWATH-MS
Source: Cell Syst. 2020 Feb 26;10(2):133–155.e6. doi: 10.1016/j.cels.2020.01.001 (PMC7042714; doi:10.1016/j.cels.2020.01.001)

Q8NEY1 | NAV1_HUMAN | NAV1 KIAA1151 KIAA1213 POMFIL3 STEERIN1
Monomer MW [kDa]: 202.472 Monomer expected elution fraction: 34

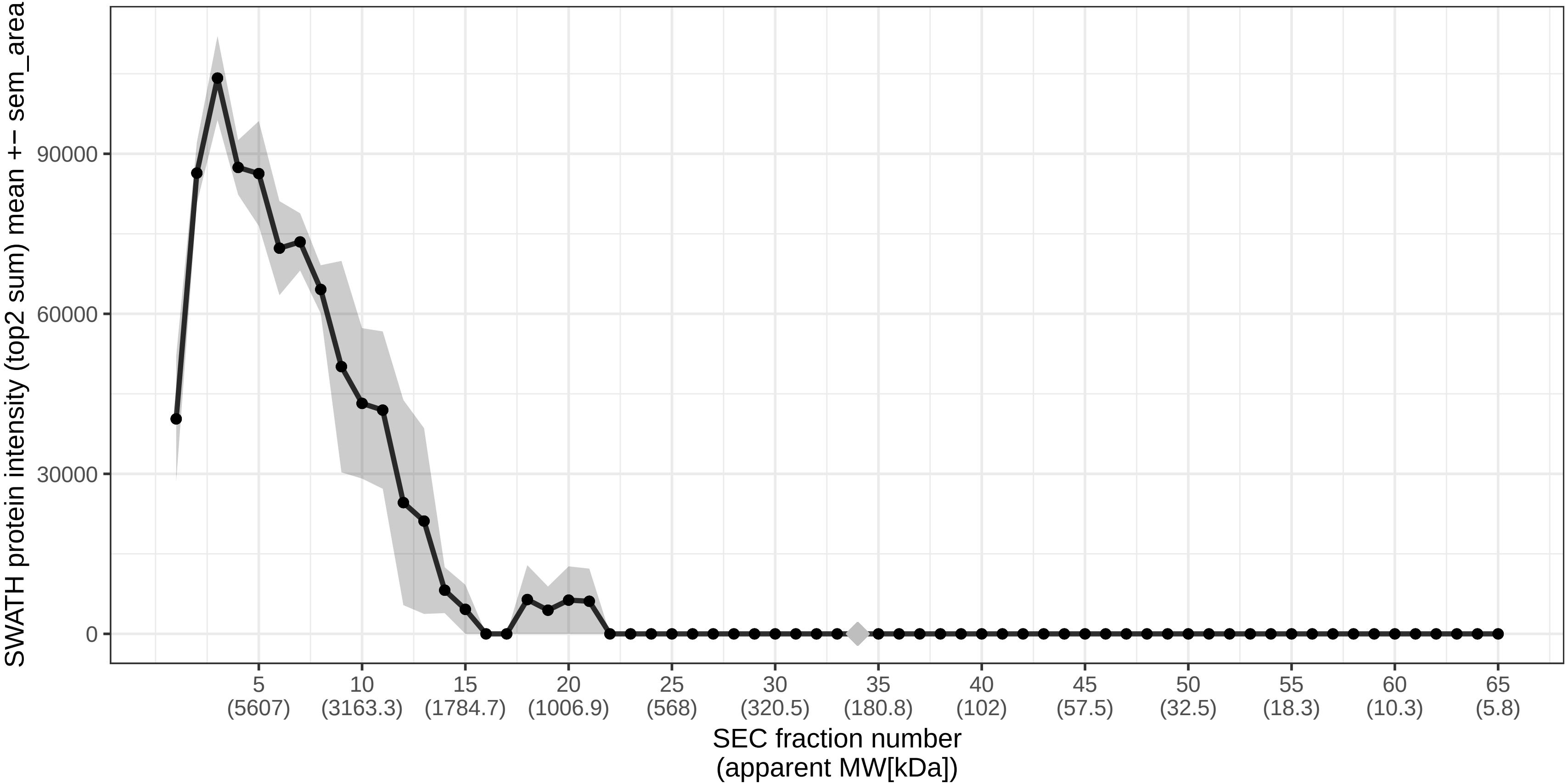

Supplement: Data S1. SEC-SWATH-MS Protein Chromatograms, Related to Figure 1 [file mmc6.zip › SECchrom_Q8NEY1_NAV1_HUMAN_NAV1_KIAA1151_KIAA1213_POMFIL3_S.pdf]
